# Aberrant *Chloride Intracellular Channel 4* Expression Is Associated With Adverse Outcome in Cytogenetically Normal Acute Myeloid Leukemia

**DOI:** 10.3389/fonc.2020.01648

**Published:** 2020-09-09

**Authors:** Sai Huang, Zhi Huang, Ping Chen, Cong Feng

**Affiliations:** ^1^Department of Hematology, First Medical Center, Chinese PLA General Hospital, Beijing, China; ^2^School of Electrical and Computer Engineering, Purdue University, West Lafayette, IN, United States; ^3^Department of Emergency, First Medical Center, Chinese PLA General Hospital, Beijing, China

**Keywords:** *CLIC4*, expression, prognostic biomarker, CN-AML, adverse outcome

## Abstract

**Background and Methods:** Acute myeloid leukemia (AML), which starts in the bone marrow, is a group of hematopoietic stem cell disorders. Chloride intracellular channel 4 (CLIC4) is regulated by p53, c-Myc, and TGF-β. It induces the NF-κB-dependent activation of HIF (hypoxia-inducible factor) and participates in tumor growth through its microenvironmental function. However, its prognostic value in AML remains unclear, as well as its co-expression biomarkers. In this study, we evaluated the prognostic significance of *CLIC4* expression using two independent large cohorts of cytogenetically normal AML (CN-AML) patients. Multivariable analysis and multi-omics analysis with weighted correlation network analysis (WGCNA) in the CN-AML group were also presented. Based on *CLIC4* and its related genes, microRNA–target gene interaction network analysis and downstream gene ontology analysis were performed to unveil the complex functions behind *CLIC4*.

**Results:** We demonstrated that the overexpression of *CLIC4* was notably associated with unfavorable outcome in the two independent cohorts of CN-AML patients [overall survival (OS) and event-free survival (EFS): *P* < 0.0001, *n* = 185; OS: *P* = 0.016, *n* = 232], as well as in the European LeukemiaNet (ELN) Intermediate-I group (OS: *P* = 0.015, EFS: *P* = 0.012, *n* = 115), the National Comprehensive Cancer Network Intermediate Risk AML group (OS and EFS: *P* < 0.0001, *n* = 225), and the non-M3 AML group (OS and EFS: *P* < 0.0001, *n* = 435). Multivariable analysis further validated *CLIC4* as a high-risk factor in the CN-AML group. Multi-omics analysis presented the overexpression of *CLIC4* as associated with the co-expression of the different gene sets in leukemia, up/downregulation of the immune-related pathways, dysregulation of microRNAs, and hypermethylation around the CpG islands, in open sea regions, and in different gene structural fragments including TSS1500, gene body, 5′UTR region, 3′UTR region, and the first exon. By further performing WGCNA on multi-omics data, certain biomarkers that are co-expressed with *CLIC4* were also unveiled.

**Conclusion:** We demonstrated that *CLIC4* is a novel, potential unfavorable prognosticator and therapeutic target for CN-AML. As having a key role in CN-AML, the interactions between *CLIC4* and other genomics and transcriptomics data were confirmed by performing microRNA–target gene interaction network analysis and gene ontology enrichment analysis. The experimental result provides evidence for the clinical strategy selection of CN-AML patients.

## Introduction

Acute myeloid leukemia (AML) is a genetically and clinically heterogeneous hematopoietic malignancy. The outcome of AML patients is generally poor and is strongly influenced by gene mutations and chromosomal alterations (1). Currently, the prognostic stratification is based on gene sequencing and cytogenetic analysis. It is used clinically to guide treatment decisions in AML patients with chromosomal abnormalities, which comprises 55–60% of all AML cases (2). However, the risk stratification guidelines for the remaining 40–45% AML patients, who lack detectable chromosomal alternations and are classified as cytogenetically normal AML (CN-AML) patients, are still not well understood (3). For CN-AML patients, the outcomes are widely diverse (4). According to the 2017 European LeukemiaNet (ELN) AML risk stratification, it is entirely determined by common gene mutations such as *NPM1*, *ASXL1*, *TP53*, *RUNX1*, *FLT3-ITD*, double mutation of *CEBPA*, etc. (5). Although the clinical validation of these molecular markers has added a great deal to the prognostic stratification of CN-AML, continuing to elucidate the biological characteristics is vital to guide treatment and provide novel targets for therapies. Accurately predicting the therapy outcome and establishing a precise risk stratification have therefore become crucial for CN-AML.

Over the past decade, the biological and physiological role for ion channels in tumor development has been widely studied. Ion channels are integral membrane proteins preserving the normal physiology of cells and regulating many cellular, organellar, and physiological processes. Evidence reveals the multitask possibility of the participation of ion channel proteins in the tumorigenesis and leukemogenesis process (6). The abnormal expression and function of ion channel proteins can contribute to the development, metastasis, and drug resistance of leukemia (7). Many investigations have indicated that the overexpression of some ion channel proteins is related to poor prognosis. Therefore, ion channels could be considered as new biomarkers in leukemia diagnosis and treatment (8).

Chloride intracellular ion channels (CLICs), a novel class of intracellular organelle ion channels, are now being widely surveyed to explore their role in tumor biology. When overexpressed heterologously, the CLIC protein family is able to generate an outwardly rectifying chloride channel activity (9). Among the CLIC protein family (CLIC1–CLIC6), several studies have focused on the role of chloride intracellular channel 4 (CLIC4) protein. In soluble form, *CLIC4* is involved in p53- and c-myc-mediated apoptosis and transforming growth factor beta (TGF-β) signaling modulation in multiple cell types (10). *CLIC4* has been proven to be involved in multiple cellular functions, including the regulation of cell proliferation, differentiation, angiogenesis, and apoptosis (11). Studies implied that *CLIC4* also induced the NF-κB-dependent activation of HIF (hypoxia-inducible factor) and participated in tumor growth through its microenvironmental function (11, 12). Subsequent studies found that *CLIC4* might be associated with adverse outcomes in colorectal cancer and esophageal squamous cell carcinoma (13, 14). Specifically, an elevated expression of *CLIC4*, a common trait of many cancers and a marker of malignant progression, was found to be associated with the activation of the Arf6 and NF-κB signaling pathway (15). However, its prognostic value in AML remains unclear.

Here, we presented *CLIC4* as a novel, potential unfavorable prognosticator and therapeutic target for CN-AML. We also explored *CLIC4*-associated genomic and epigenomic patterns to further elucidate its key role in CN-AML. Our study represented direct evidences for *CLIC4* expression as a prognostic biomarker in multiple CN-AML datasets. We also brought insights into CN-AML risk stratification and potential therapeutic targets in leukemia prognosis and treatment.

## Materials and Methods

### Patients and Treatment

This study was approved by local institutional review boards and written informed consent was provided for all patients according to the Declaration of Helsinki.

The first cohort was derived from the whole AML cohort (GSE6891) with 185 primarily untreated CN-AML patients (median age = 47 years, range = 16–60 years) diagnosed and collected at Erasmus University Medical Center from 1990 to 2008. Conventional cytogenetics examined more than 20 metaphases from bone marrow (BM) samples to determine normal karyotype.

The second independent cohort was derived from the multicenter AMLCG-1999 trial of the German AML Cooperative Group (GSE12417) with 163 primarily untreated CN-AML patients (median age = 57.5 years, range = 17–83 years). This cohort was used to validate the results of the first cohort.

All the clinical, cytogenetic, molecular, and microarray data of these CN-AML patients were obtained from the Gene Expression Omnibus (GEO) database^1^. Overall survival (OS) and event-free survival (EFS) data were obtained from the GEO database and their publications.

### Microarray Analysis

Gene expression was derived from five GEO datasets—GSE9476, GSE30029, GSE6891, GSE12417, and GSE71014—using Affymetrix Human Genome 133 plus 2.0 and U133A gene chips. All datasets were normalized as previously described (16). Profiles of the messenger RNAs (mRNAs) and microRNAs and the genome-wide methylation data of 73 CN-AML patients were derived from The Cancer Genome Atlas (TCGA). Expression data of mRNA and microRNA were obtained by RNA sequencing, whereas methylation data was obtained by Illumina Infinium 450K BeadChips. One hundred and eighty-five CN-AML patients from GSE6891 (435 AML patients, no M3) were divided into four quartiles to choose the appropriate subdivision cutoff value. The expression of *CLIC4* represented a normal distribution. A significant distinction was shown along the median value (Supplementary Figures S1A–C). Thus, the median value of *CLIC4* expression was used to divide patients into *CLIC4*^high^ and *CLIC4*^low^ groups. The expression levels of *ERG*, *DNMT3A*, and other genes were examined using the same strategy.

### Statistical Analysis

The primary endpoints were OS and EFS. The former was defined as the time from diagnosis to death by any cause, while the latter was defined as the time from date of diagnosis to the first event including failure to achieve complete remission (CR), relapse, or death. Between-group comparisons of OS and EFS were performed by the Kaplan–Meier and log-rank tests. The association of *CLIC4* expression and clinical factors including age, sex, and FAB classification was analyzed using Fisher’s exact and Wilcoxon rank-sum tests for categorical and continuous variables, respectively. The impacts of *CLIC4* expression on OS and EFS were assessed by multivariable hazard models. Multiple hypothesis correction (false discovery rate, FDR) as well as Student’s *t*-test were used to validate differences in the mRNA, microRNA, and DNA methylation profiles between the *CLIC4*^high^ and *CLIC4*^low^ groups (17). All analyses were performed by R3.6.3 and its related software packages.

### Transcriptome, Gene Ontology, and Functional Enrichment Analysis

The differentially expressed genes analyzed in the genome-wide gene-expression profiles were then exported to ToppGene Suite^2^ (18). Functional enrichment analysis was performed using ToppGene Suite, which is widely accepted for gene ontology enrichment analysis. Hypergeometric distribution with Bonferroni correction was used as the standard method to determine significance. A FDR < 0.05 was considered to be significantly enriched for these analyses (19).

### Weighted Correlation Network Analysis for Multi-Omics Data

Weighted correlation network analysis (WGCNA), one of the popular gene co-expression network identification tools (20), was adopted to further unveil the *CLIC4*-associated biomarkers across the mRNA, microRNA, and DNA methylation profiles. Gene filtering was performed with the TSUNAMI web portal (21). The target gene co-expression module, where *CLIC4* was located at, was performed with downstream enrichment analysis.

## Results

### Overexpression of *CLIC4* in AML Patients

**FIGURE 1:**
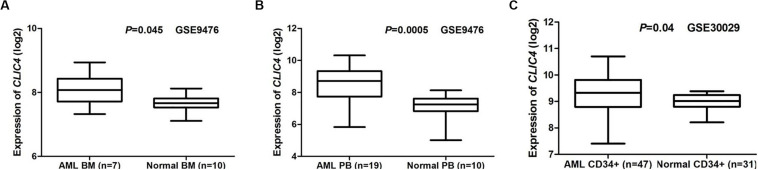
Different expressions of *CLIC4*. **(A)** AML bone marrow samples (*n* = 7) vs. normal bone marrow samples (*n* = 10). **(B)** AML peripheral blood samples (*n* = 19) vs. normal peripheral blood samples (*n* = 10). **(C)** AML CD34^+^ samples (*n* = 47) and normal CD34^+^ samples (*n* = 31). AML, acute myeloid leukemia.

Two public GEO microarray datasets (GSE9476 and GSE30029) were used to compare *CILC4* expression in the BM, peripheral blood (PB), and CD34^+^ cell samples from AML patients and healthy donors. The results showed that *CLIC4* was significantly overexpressed in AML BM than in normal BM (seven AML BM vs. 10 normal BM, GSE9476, *P* = 0.045) (Figure 1A), which was validated by PB in the same assay (19 AML PB vs. 10 normal PB, *P* = 0.0005) (Figure 1B). CD34^+^ cells derived from AML patients further validated *CLIC4* overexpression in AML (47 AML CD34^+^ vs. 31 normal CD34^+^, GSE30029, *P* = 0.04) (Figure 1C). All results revealed that CLIC4 expression was markedly upregulated in AML patients.

### Clinical and Molecular Characteristics Between the *CLIC4*^low^ and *CLIC4*^high^ Groups in AML Patients

In the cohort of 185 CN-AML patients from the GSE6891 dataset, the *CLIC4*^high^ group tends to have fewer *NPM1* mutations (*P* = 0.0209) and more *FLT3-ITD* patients (*P* < 0.0001), while no significant differences between the *CLIC4*^low^ and *CLIC4*^high^ groups were observed in the *CEBPA* and *N-ras/K-ras* mutation groups (*P* = 0.5949 and *P* = 0.195, respectively). Furthermore, the *CLIC4*^high^ group was more likely to overexpress *BAALC*, *MAPKBP1*, *RUNX1*, and *TCF4*, which were proven to contribute to adverse outcomes in CN-AML. Interestingly, patients in the *CLIC4*^high^ group were more likely to relapse than those in the *CLIC4*^low^ group (Table 1). These data indicated that *CLIC4* overexpression was possibly related to adverse outcomes in CN-AML.

**TABLE 1:** Clinical and molecular characteristics of 185 cytogenetically normal acute myeloid leukemia (CN-AML) patients in the *CLIC4*^low^ and *CLIC4*^high^ groups.

Characteristics	*CLIC4*		*U*/χ ^2^	*P*
	Low expression group	High expression group		
	(*n* = 92)	(*n* = 93)		
Median age, years (range)	47.5(16−60)	46(17−60)	4112	0.6483
**Gender, *n* (%)**			1.953	0.1623
Male	51 (55.4)	42 (45.7)		
Female	41 (44.6)	51 (54.8)		
**FAB subtype, *n* (%)**				
M0	2 (2.17)	2 (1.08)	0.0001	0.9913
M1	23 (25.00)	29 (31.18)	0.8749	0.3496
M2	14 (15.22)	22 (23.65)	2.101	0.1472
M4	15 (16.30)	13 (13.98)	0.1948	0.659
M5	28 (30.44)	24 (25.81)	0.4904	0.4838
M6	3 (3.26)	0 (0.00)	3.083	0.0791
Other	7 (7.61)	4 (4.30)	0.9048	0.3415
***CEBPA*, *n* (%)**			1.039	0.5949
Single mutation	3 (3.26)	3 (3.22)		
Double mutation	11 (11.96)	7 (7.53)		
Wild-type	78 (84.78)	82 (89.25)		
***NPM1*, *n* (%)**			5.338	0.0209
Mutation	60 (65.22)	45 (48.39)		
Wild-type	32 (34.78)	48 (51.61)		
***FLT3-ITD*, *n* (%)**			15.72	< 0.0001
Presence	25 (27.17)	52 (55.91)		
Absence	67 (72.83)	44 (44.09)		
***N-ras*/*K-ras***			1.68	0.195
Mutation	11 (11.96)	6 (6.45)		
Wild-type	81 (88.04)	87 (93.55)		
***ERG* expression, *n* (%)**			0.2653	0.6065
High	48 (52.17)	45 (48.39)		
Low	44 (27.83)	48 (51.61)		
***BAALC* expression, *n* (%)**			5.885	0.0153
High	38 (41.30)	55 (59.14)		
Low	54 (58.70)	38 (40.86)		
***WT1* expression, *n* (%)**			0.1349	0.7135
High	45 (48.91)	48 (51.61)		
Low	47 (51.09)	45 (48.39)		
***DNMT3A* expression, *n* (%)**			0.9161	0.3385
High	50 (54.35)	44 (47.31)		
Low	42 (45.65)	49 (52.69)		
***DNMT3B* expression, *n* (%)**			2.383	0.1227
High	41 (44.57)	52 (55.91)		
Low	51 (55.43)	41 (44.09)		
***MAPKBP1* expression, *n* (%)**			5.885	0.0153
High	38 (41.30)	55 (59.14)		
Low	54 (58.70)	38 (40.86)		
***ITPR2* expression, *n* (%)**			2.383	0.1227
High	41 (44.57)	52 (55.91)		
Low	51 (55.43)	41 (44.09)		
***ATP1B1* expression, *n* (%)**			15.18	< 0.0001
High	33 (35.87)	60 (64.52)		
Low	59 (64.13)	33 (35.48)		
***RUNX1* expression, *n* (%)**			9.085	0.0026
High	36 (39.13)	57 (61.29)		
Low	56 (60.87)	36 (38.71)		
***TCF4* expression, *n* (%)**			17.56	< 0.0001
High	32 (34.78)	61 (65.59)		
Low	60 (65.22)	32 (34.41)		
**Relapse**			10.77	0.001
Yes	55 (59.78)	76 (81.72)		
No	37 (40.22)	17 (18.28)		

### Overexpression of *CLIC4* Was Associated With Unfavorable Outcomes in AML Patients

**FIGURE 2:**
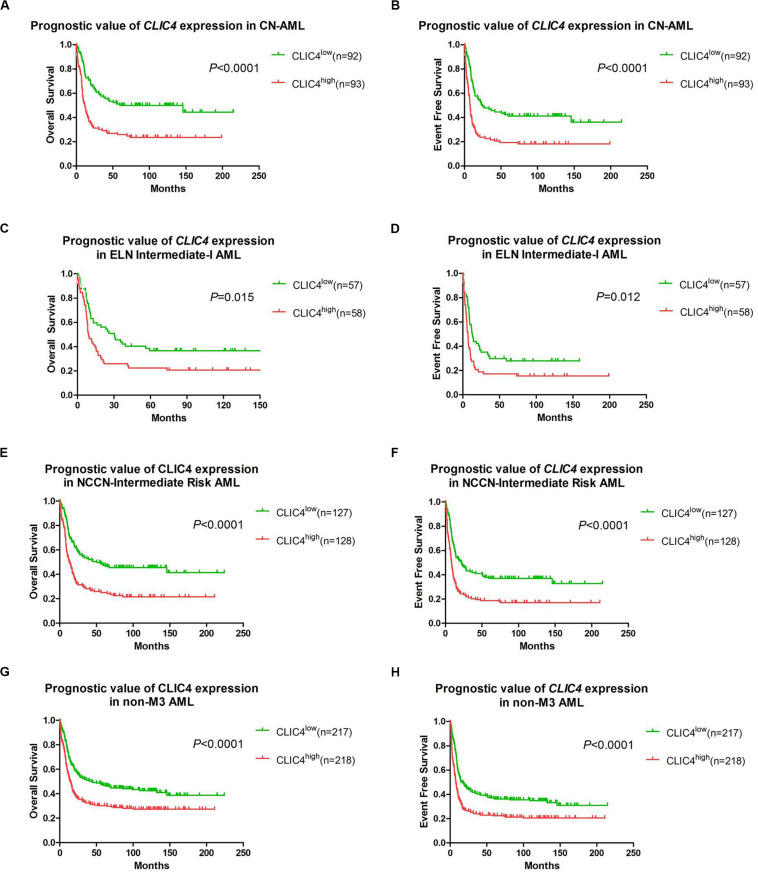
The prognostic significance of *CLIC4* expression in acute myeloid leukemia (AML) patients. Overall survival and event-free survival in 185 CN-AML patients **(A,B)**, 115 ELN Intermediate-I patients **(C,D)**, 255 NCCN-Intermediate Risk AML patients **(E,F)**, and 435 non-M3 AML patients **(G,H)**.

In order to further validate the prognostic role of *CLIC4*, survival analyses were carried out in the cohort of 185 CN-AML patients and 115 ELN Intermediate-I patients derived from GSE6891. The results showed that the *CLIC4*^high^ group had notably shorter OS and EFS in CN-AML patients (*CLIC4*^low^ vs. *CLIC4*^high^: median OS = 59.4 vs. 11.5 months, *P* < 0.0001; median EFS: 23.95 vs. 7.56 months, *P* < 0.0001) (Figures 2A,B) as well as in the ELN Intermediate-I category (*CLIC4*^low^ vs. *CLIC4*^high^: median OS = 30.39 vs. 8.9 months, *P* = 0.015; median EFS = 13.01 vs. 6.83 months, *P* = 0.012) (Figures 2C,D). To further examine the impact of *CLIC4* expression on survival, multivariable Cox proportional hazard models were constructed using multiple variables in the above cohorts (Table 2). For the cohort of 185 CN-AML patients, the *CLIC4*^high^ group had 1.3 times higher risk of EFS (*P* < 0.01) and 1.36 higher risk of OS (*P* < 0.01). Other adverse factors included the presence of *FLT3-ITD* (*P* < 0.01 for both EFS and OS), the presence of *FLT3-TKD* (*P* < 0.01 for OS), and mutated *N-ras*/*K-ras* (*P* < 0.05 for EFS). For the cohort of 115 ELN Intermediate-I patients, the *CLIC4*^high^ group had 1.23 times higher risk of EFS (*P* < 0.05) and 1.31 higher risk of OS (*P* < 0.01). Other adverse factors included the presence of *FLT3-TKD* (*P* < 0.01 for OS) and mutated *IDH1/IDH2* (*P* < 0.05 for OS). These results indicated that the overexpression of *CLIC4* was an independent risk factor for both OS and EFS in CN-AML patients and in the ELN Intermediate-I category.

**TABLE 2:** Multivariable analysis of overall survival (OS) and event-free survival (EFS) in 185 cytogenetically normal acute myeloid leukemia (CN-AML) and 115 European LeukemiaNet (ELN) Intermediate-I patients.

Variables	EFS			OS		
	HR	95% CI	*P-*value	HR	95% CI	*P-*value
**All CN-AML, *n* = 185**						
*CLIC4* expression, high vs. low	1.30	1.12–1.52	< 0.01	1.36	1.15–1.60	< 0.01
Risk, poor vs. non-poor	1.05	0.24–4.65	0.95	1.99	0.45–8.82	0.36
Age, per 10-year increase	1.05	0.91–1.22	0.52	1.09	0.92–1.27	0.32
Gender, male vs. female	1.21	0.85–1.73	0.30	1.39	0.95–2.02	0.09
*CEBPA*, mutated vs. wild-type	0.72	0.39–1.31	0.28	0.83	0.43–1.58	0.57
*NPM1*, mutated vs. wild-type	0.72	0.46–1.11	0.13	0.78	0.50–1.22	0.28
FLT3-ITD, presented vs. others	1.82	1.21–2.75	< 0.01	1.72	1.12–2.64	< 0.01
*FLT3-TKD*, presented vs. others	1.52	0.86–2.68	0.15	1.96	1.09–3.52	< 0.01
*IDH1*/*IDH2*, mutated vs. wild-type	0.82	0.52–1.31	0.41	0.78	0.48–1.27	0.32
*N-ras*/*K-ras*, mutated vs. wild-type	2.20	1.17–4.13	< 0.05	1.68	0.86–3.28	0.13
**ELN Intermediate-I, *n* = 115**						
*CLIC4* expression, high vs. low	1.23	1.03–1.46	< 0.05	1.31	1.08–1.59	< 0.01
Risk, poor vs. non-poor	1.12	0.24–5.27	0.89	1.62	0.35–7.52	0.53
Age, per 10-year increase	1.12	0.93–1.34	0.23	1.17	0.96–1.41	0.12
Gender, male vs. female	1.29	0.84–2.00	0.25	1.57	0.99–2.49	0.05
*CEBPA*, mutated vs. wild-type	1.20	0.15–9.55	0.86	1.76	0.22–14.39	0.60
*NPM1*, mutated vs. wild-type	0.78	0.40–1.52	0.47	1.20	0.60–2.39	0.60
*FLT3-ITD*, presented vs. others	1.50	0.75–3.04	0.26	0.99	0.48–2.04	0.97
*FLT3-TKD*, presented vs. others	2.03	0.81–5.08	0.13	3.99	1.59–10.00	< 0.01
*IDH1*/*IDH2*, mutated vs. wild-type	0.61	0.33–1.12	0.11	0.51	0.27–0.98	< 0.05
*N-ras*/*K-ras*, mutated vs. wild-type	1.55	0.59–4.06	0.37	1.29	0.50–3.31	0.60

Additional survival analyses were carried out in the cohort of 255 NCCN Intermediate Risk patients and 435 non-M3 AML patients. Similarly, shorter OS and EFS were shown in the *CLIC4*^high^ group both in the NCCN Intermediate Risk category (*CLIC4*^low^ vs. *CLIC4*^high^: median OS = 50.4 vs. 13.06 months, *P* < 0.0001; median EFS = 21.85 vs. 8.35 months, *P* < 0.0001) (Figures 2E,F) and in non-M3 AML patients (*CLIC4*^low^ vs. *CLIC4*^high^: median OS = 43.6 vs. 14.49 months, *P* < 0.0001; median EFS = 16.66 vs. 7.69 months, *P* < 0.0001) (Figures 2G,H). Another independent cohort of 163 *de novo* CN-AML patients (GSE12417) was further studied, which showed that *CLIC4*^high^ was significantly associated with shorter OS (*CLIC4*^low^ vs. *CLIC4*^high^: median OS = 14.73 vs. 11 months, *P* = 0.0032) (Supplementary Figure S2).

### Genome-Wide Gene Expression Profiles Associated With *CLIC4* Expression

**FIGURE 3:**
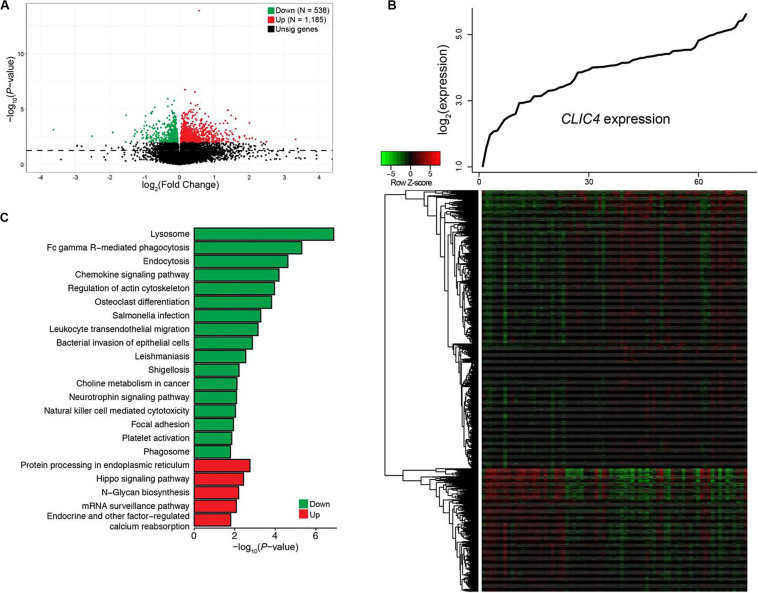
Genome-wide genes/cell signaling pathways associated with *CLIC4* expression. **(A)** Volcano plot of the genome-wide gene profiles between the *CLIC4*^high^ and *CLIC4*^low^ groups. **(B)** Gene expression heatmap. The *top curve* indicates the expression distributions of *CLIC4* in 73 cytogenetically normal acute myeloid leukemia (CN-AML) samples. **(C)** Cell signaling pathways associated with *CLIC4* expression.

To gain a better understanding of the biological value of *CLIC4* in leukemogenesis, we derived the *CLIC4*-associated gene expression profiles from high-throughput sequencing in the 73 CN-AML patients from the TCGA dataset. A total of 1,185 upregulated 538 downregulated genes were found to be significantly associated with the *CLIC4*^high^ group (FDR-adjusted *P* < 0.01) (Figures 3A,B). The 1,185 upregulated genes were further exported to the ToppGene Suite. A total of 105 genes were found to be significantly enriched in chronic lymphocytic leukemia (CLL) (overlap = 105/633, *P* = 6.461e-32) (Supplementary Table S1) (22). Another set of 153 genes were found to be enriched in CD34^+^ cells isolated from the BM of patients with chronic myeloid leukemia (CML) compared to those from normal donors (overlap = 153/1,399, *P* = 2.400e-25) (Supplementary Table S2) (23). Besides, a total of 53 genes were significantly upregulated in cancer stem cells isolated from mammary tumors compared to those from the non-tumorigenic cells (overlap = 53/432, *P* = 3.249e-11) (Supplementary Table S3) (24). Interestingly, 19 genes were significantly enriched in a probe-set gene-expression signature that predicted survival in CN-AML (overlap = 19/62, *P* = 1.406e-11) (Supplementary Table S4) (16). The top Gene Ontology (GO) biological process term was protein ubiquitination (GO:0016567; overlap = 69/852, *P* = 4.282e-5). Five hundred and thirty-eight downregulated genes were analyzed as previously described. A total of 23 genes were among the top 100 probe sets for pediatric AML subtypes with chimeric MLL fusions (overlap = 23/81, *P* = 1.033e-19) (Supplementary Table S5). The top GO biological process terms were myeloid cell activation involved in immune response (GO:0002275; overlap = 82/557, *P* = 1.563e-40) and myeloid leukocyte-mediated immunity (GO:0002444; overlap = 82/568, *P* = 7.022e-40).

Cell signal pathways associated with *CLIC4*, analyzed from the *MSigDB* data, further provided new insights into the leukemogenesis of CN-AML. A total of five upregulated and 17 downregulated pathways showed significant associations with the *CLIC4*^high^ group (*P* < 0.05; Figure 3C). The upregulated pathway included the hippo signaling pathway, an important canonical oncogenic pathway. However, the downregulated pathways were mostly immune activation pathways such as natural killer cell-mediated cytotoxicity, as well as chemokine and neurotrophin signaling pathways. These dysregulated gene expressions and signaling pathways might provide insights into the involvement between *CLIC4* and leukemogenesis of CN-AML.

### Genome-Wide MicroRNA Profiles Associated With *CLIC4* Expression

**FIGURE 4:**
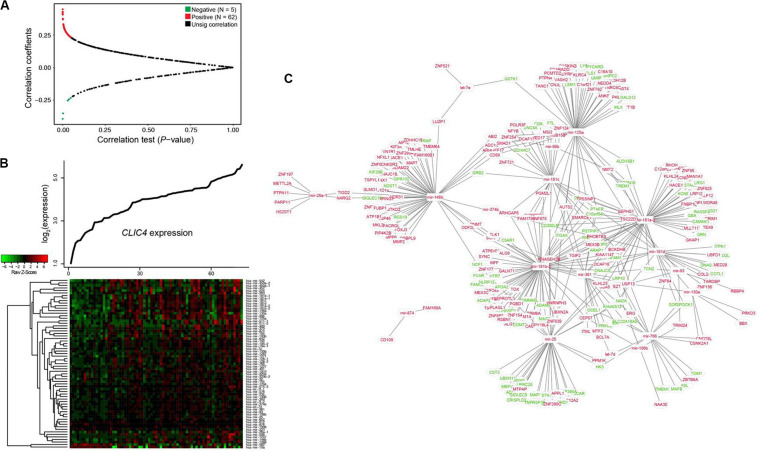
Interaction network analysis of genome-wide microRNAs and their target genes associated with *CLIC4* expression. **(A)** Volcano plot of the genome-wide microRNA profiles between the *CLIC4*^high^ and *CLIC4*^low^ groups. **(B)** MicroRNA expression heatmap. The *top curve* indicates the expression distributions of *CLIC4* in 73 cytogenetically normal acute myeloid leukemia (CN-AML) samples. **(C)** MicroRNA–mRNA interaction network analysis (*red*: upregulation; *green*: downregulation; *rectangle*: microRNAs; *ellipse*: mRNAs).

Investigation of the dysregulated microRNAs could be used to determine the extent of heterogeneity in AML. Therefore, the *CLIC4*-associated genome-wide microRNA profiles in 73 CN-AML patients from the TCGA dataset were analyzed. A total of 67 microRNAs were significantly correlated with *CLIC4* expression, including 62 positive and five negative microRNAs (*P* < 0.05; Figures 4A,B). Positively correlated microRNAs included miR-25, miR-106b, miR-146a, miR-146b, miR-155, miR-181a-1, miR-181a-2, miR-181b-1, miR-181b-2, miR-181c, miR-181d, etc. MiR-25 was found to be overexpressed in the c-kit subgroup and involved in leukemogenesis in pediatric AML. MiR-106b was significantly upregulated in refractory and relapsed pediatric MLL-rearranged AML (25). MiR-146a and miR-146b were abundant in t(8;21) AML, while miR-155 were abundant in *FLT3-ITD* AML (26). The miR-181 family (miR-181a-1, miR-181a-2, miR-181b-1, miR-181b-2, miR-181c, and miR-181d) is regarded as oncogenic in several cancers and its upregulated expression predicted unfavorable outcomes in AML (27). The negatively correlated microRNAs were miR-10a, miR-146b, miR-1266, etc. MiR-10a was found downregulated in hematological tumor cell lines by targeting *BCL6* (28). MiR-146b could delay the aggressiveness and progression of T-ALL (29). miR-1266 was downregulated in childhood T-ALL and might be used to distinguish childhood ALL subtypes (30).

Interaction network analysis was conducted to further validate the microRNA–target gene correlation results (Figure 4C). Many tumor repressors were found to be the targets of upregulated microRNAs, contributing to worse outcomes. For example, *GPR132*, targeted by miR-146b, could exert growth inhibitory and apoptotic effects in AML, ALL, and CML cell lines (31). *PYCARD*, targeted by miR-125a, was shown to be downregulated to different extents in a wide spectrum of human cancers and prevented tumor cells from undergoing apoptosis (32). These results helped explain the reason why *CLIC4* acted as an adverse biomarker and provided a comprehensive view of its molecular mechanisms.

### Genome-Wide Methylation Profiles Associated With *CLIC4* Expression

**FIGURE 5:**
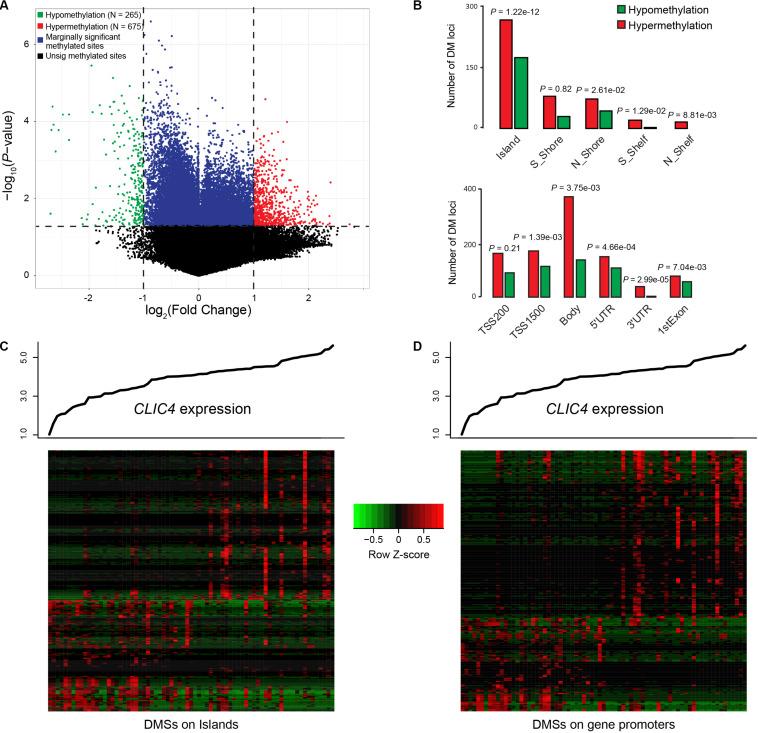
Genome-wide methylation profiles associated with *CLIC4* expression. **(A)** A total of 265 hypomethylation and 675 hypermethylation differential methylated regions were discovered from the comparison between the *CLIC4*^high^ and *CLIC4*^low^ groups. **(B)** More hypermethylation differential methylated regions were found around the CpG islands and in open sea regions, while more hypermethylated differential methylated regions lie on the body and the 3′UTR region. **(C,D)** Heatmaps showed *CLIC4*-associated differential methylated regions enriched on islands and gene promoter regions.

Differential methylated regions (DMR) were further derived to investigate the different methylation patterns between the *CLIC4*^low^ and *CLIC4*^high^ groups in 73 CN-AML patients from the TCGA dataset. Firstly, a total of 265 hypomethylation and 675 hypermethylation DMRs were derived from the comparison between the *CLIC4*^low^ and *CLIC4*^high^ groups [*P* < 0.05, | log2(FC)| > 1] (Figure 5A). Secondly, position distribution around the CpG islands was compared in these abnormal DMRs. More hypermethylation DMRs were around the CpG islands (Island: *P* = 1.22e-12, N_Shore: *P* = 2.61e-02) and in open sea regions (S_Shelf: *P* = 1.29e-02, N_Shelf: *P* = 8.81e-03) (upper subfigure of Figure 5B). Thirdly, the position distribution of the different gene structural fragments was presented, showing hypermethylated DMRs lying on TSS1500, gene body, the 5′UTR region, 3′UTR region, and first exon (*P* = 1.39e-03, *P* = 3.75e-03, *P* = 4.66e-04, *P* = 2.99e-05, and *P* = 7.04e-03, respectively) (lower subfigure of Figure 5B). Finally, *CLIC4*-related DMRs enriched on islands and gene promoter regions were presented in heatmaps (Figures 5C,D).

### Identifying *CLIC4*-Associated Biomarkers With Multi-Omics WGCNA

As the genomics and transcriptomics data remain noises, it is necessary to filter out some genes before the multi-omics co-expression analysis by WGCNA. We filtered out mRNA genes with the lowest 20% means and variances of expression values, filtered out microRNA genes with the lowest 20% means and variances of expression values, and filtered out methylation profiles with the lowest 80% means and variances of expression values *via* the TSUNAMI web portal. We ended up with 13,082 mRNA genes, 681 microRNA genes, and 15,843 methylation profiles. We considered them as the combined multi-omics biomarkers (29,606 in total) and performed biomarker-wise normalization (norm = 1 for all biomarkers) before performing the WGCNA.

**FIGURE 6:**
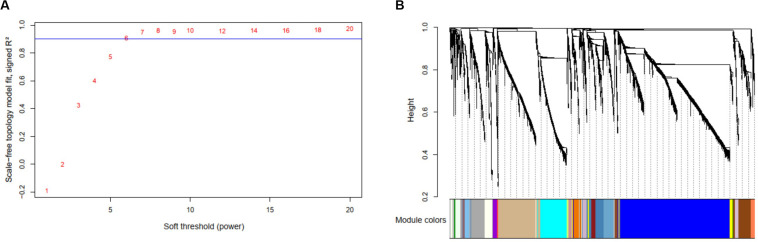
Results of the multi-omics analysis with weighted correlation network analysis (WGCNA). **(A)** Scale-free topology plot to determine soft threshold (power). **(B)** The identified 136 WGCNA co-expression modules and the dendrogram.

By selecting the soft threshold (power) = 6 (Figure 6A), minimum module size = 10, and merge cut height of the dendrogram = 0.25, 136 co-expressed modules were identified (Figure 6B). The gene of interest *CLIC4* is located at the module with a dark turquoise color which contained 25 mRNA genes, five microRNAs (miR-211, miR-28, miR-511-1, miR-511-2, and miR-581), and 238 methylation profiles. The complete co-expression module list is provided in a Supplementary Material.

Enrichment analysis was further performed based on the targeted co-expression module with 25 mRNA genes (include *CLIC4*). We found that those genes were enriched in the chromosome location 19p13.3 (genes: *SEMA6B*, *LRG1*, and *PLIN5;* overlap = 3/25, *P* = 5.698e-4), in agreement with several existing literatures (33, 34), which further confirmed the module relationship to the AML.

## Discussion

With the increasing use of multi-omics analysis in research and clinical routine, the current prognostic genes in CN-AML will likely be expanded. The new gene list will serve as a valuable tool in advancing the individualized treatment approaches in CN-AML. Recent studies has shown that *CLIC4*, an integral component of TGF-β signaling, has been proven to either enhance or inhibit tumor growth depending on the tumor type. Downregulation of *CLIC4* could promote tumor cell growth and clonogenicity in lung cancer and gastric cancer, while its upregulation could cause tumor cell growth in squamous cell carcinomas and breast cancer (35).

Our study demonstrated a critical unfavorable prognostic role of *CLIC4* for CN-AML. Firstly, *CLIC4* was found overexpressed in AML BM and PB samples than in normal BM and PB samples, and also in AML CD34^+^ than in normal CD34^+^ samples (Figure 1). Secondly, the clinical and molecular characteristics related to *CLIC4* expression were explored in a cohort of 185 CN-AML patients. We found that the *CLIC4*^high^ group contained fewer *NPM1* mutations (*P* = 0.0209) and more *FLT3-ITD* patients (*P* < 0.0001) and remarkably high expressions of *BAALC* (*P* = 0.0153), *MAPKBP1* (*P* = 0.0153), *RUNX1* (*P* = 0.0026), and *TCF4* (*P* < 0.0001), which were proven to contribute to unfavorable outcomes in CN-AML. The *CLIC4*^high^ group also had more relapse patients than the *CLIC4*^low^ group (Table 1). Multivariable Cox proportional hazard models further indicated *CLIC4* as an independent biomarker for CN-AML (Table 2). Thirdly, survival analysis based on two relatively large and independent CN-AML cohorts (GSE6891 and GSE12417) showed that *CLIC4*^high^ was associated with adverse outcomes (Figures 2A–D). Similar results were shown in ELN Intermediate-I category, NCCN Intermediate Risk category, and non-M3 AML patients (GSE6891; Figures 2E–H). Our results might provide insights into further stratification of CN-AML, ELN Intermediate-I category, and NCCN Intermediate Risk category.

Multi-omics analysis of the genetic and epigenetic mechanisms was important in leukemogenesis, including abnormal gene expression, microRNA–target gene interaction network analysis, and downstream gene ontology analysis (18, 36). Our study further analyzed how *CLIC4* overexpression affected the prognosis in CN-AML from these aspects. We derived the *CLIC4*-associated genes, microRNAs, and cell signaling pathways. Using the ToppGene Suite, we discovered several sets of genes that were significantly co-expressed in CLL, CML, cancer stem cells, as well as probe-set signature that predicted survival in CN-AML. Dysregulated signaling pathways also provided evidence of the involvement of *CLIC4* in multiple cellular pathways (Figure 3).

MicroRNA-involved mechanisms related to the prognostic role of *CLIC4* were further studied. Those that were known as oncogenic or tumor suppressive were positively or negatively correlated with *CLIC4*, respectively (such as miR-181 family and miR-10a). Other synchronized changes of microRNA–target gene (such as *GPR132* targeted by miR-146b and *PYCARD* targeted by miR-125a) might promote leukemogenesis in CN-AML (Figure 4). DMRs were mostly hypermethylated around the CpG islands, in open sea regions, and in almost all gene structural fragments. Heatmaps of the *CLIC4*-related DMRs enriched on islands and gene promoter regions were also presented (Figure 5). These genes, pathways, microRNAs, microRNA–target genes, and hypermethylation DMRs might be associated with adverse prognosis of CN-AML and establish a better understanding of leukemogenesis.

Nevertheless, a multi-omics WGCNA was further performed by combining the mRNAs, microRNAs, and methylation profiles. The targeted module which contains *CLIC4* was also genetically enriched in chromosome location 19p13.3, which not only reflects some existing leukemia literatures but also suggests a potential important cytoband which deserves further analysis.

## Conclusion

In summary, we performed a large-scale multi-omics analysis of *CLIC4* in CN-AML and portrayed a hypothetical mechanism figure (Supplementary Figure S3). We provided strong evidences that *CLIC4*^high^ patients were characterized by different adverse factors and that overexpression of *CLIC4* was an adverse biomarker for CN-AML. Novel targeted therapeutic approaches and more prognostic biomarkers will be further discovered, which hold promise for improving CN-AML patients’ outcomes.

## Data Availability Statement

Publicly available datasets were analyzed in this study. This data can be found here: Gene Expression Omnibus (GSE9476, GSE30029, GSE6891, GSE12417, and GSE71014) and The Cancer Genome Atlas.

## Author Contributions

SH, ZH, and PC designed and performed the study and wrote the manuscript. ZH and CF analyzed the data and revised the manuscript. All authors reviewed the final manuscript.

## Conflict of Interest

The authors declare that the research was conducted in the absence of any commercial or financial relationships that could be construed as a potential conflict of interest.

## Footnotes

1 http://www.ncbi.nlm.nih.gov/geo

2 https://toppgene.cchmc.org/

## Supplementary Material

FIGURE S1 Median value of *CLIC4* expression as the cut-off. **(A)** Normally distribution of *CLIC4* expression. **(B)** OS and **(C)** EFS of CN-AML patients, the patients were subdivided into four quartiles based on the quartile of *CLIC4* expression.

FIGURE S2 The validation of the unfavorable outcome associated with *CLIC4*^high^ in a cohort of 163 CN-AML patients.

FIGURE S3 The hypothetical mechanism figure of *CLIC4* in CN-AML.
