# Supplementary figures and images for: Aberrant Chloride Intracellular Channel 4 Expression Is Associated With Adverse Outcome in Cytogenetically Normal Acute Myeloid Leukemia
Source: Front Oncol. 2020 Sep 9;10:1648. doi: 10.3389/fonc.2020.01648 (PMC7507859; doi:10.3389/fonc.2020.01648)

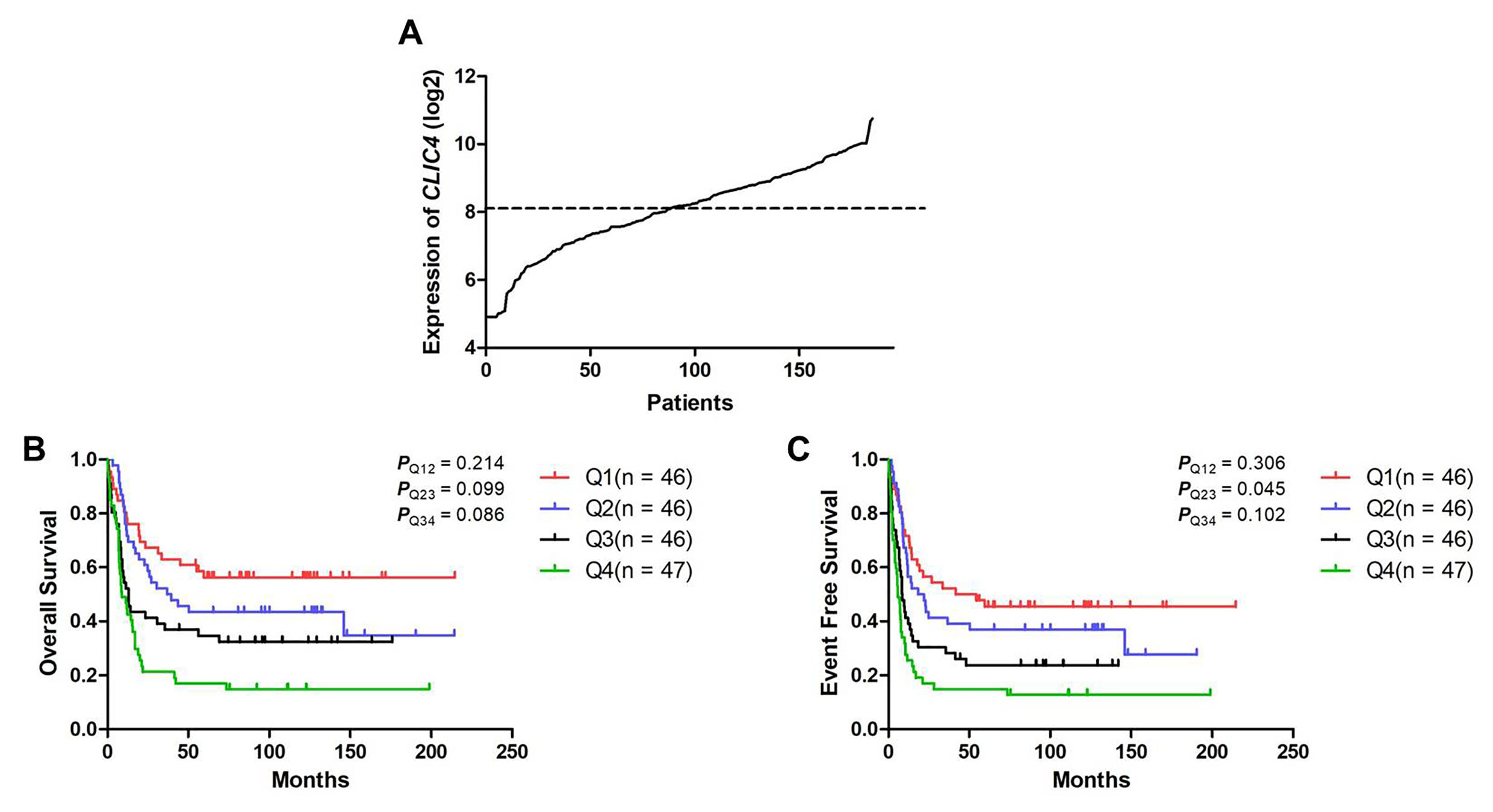

Supplement: FIGURE S1 — Median value of CLIC4 expression as the cut-off. (A) Normally distribution of CLIC4 expression. (B) OS and (C) EFS of CN-AML patients, the patients were subdivided into four quartiles based on the quartile of CLIC4 expression. [file Image_1.JPEG]

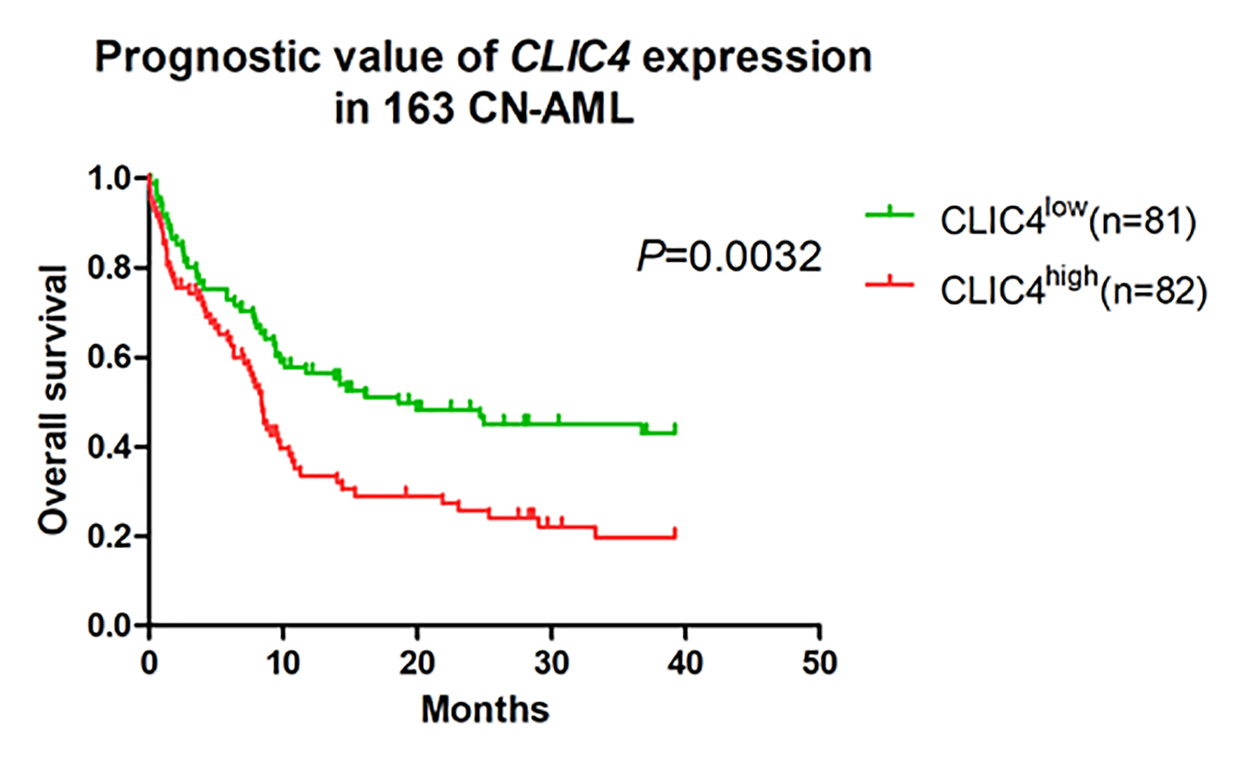

Supplement: FIGURE S2 — The validation of the unfavorable outcome associated with CLIC4high in a cohort of 163 CN-AML patients. [file Image_2.JPEG]

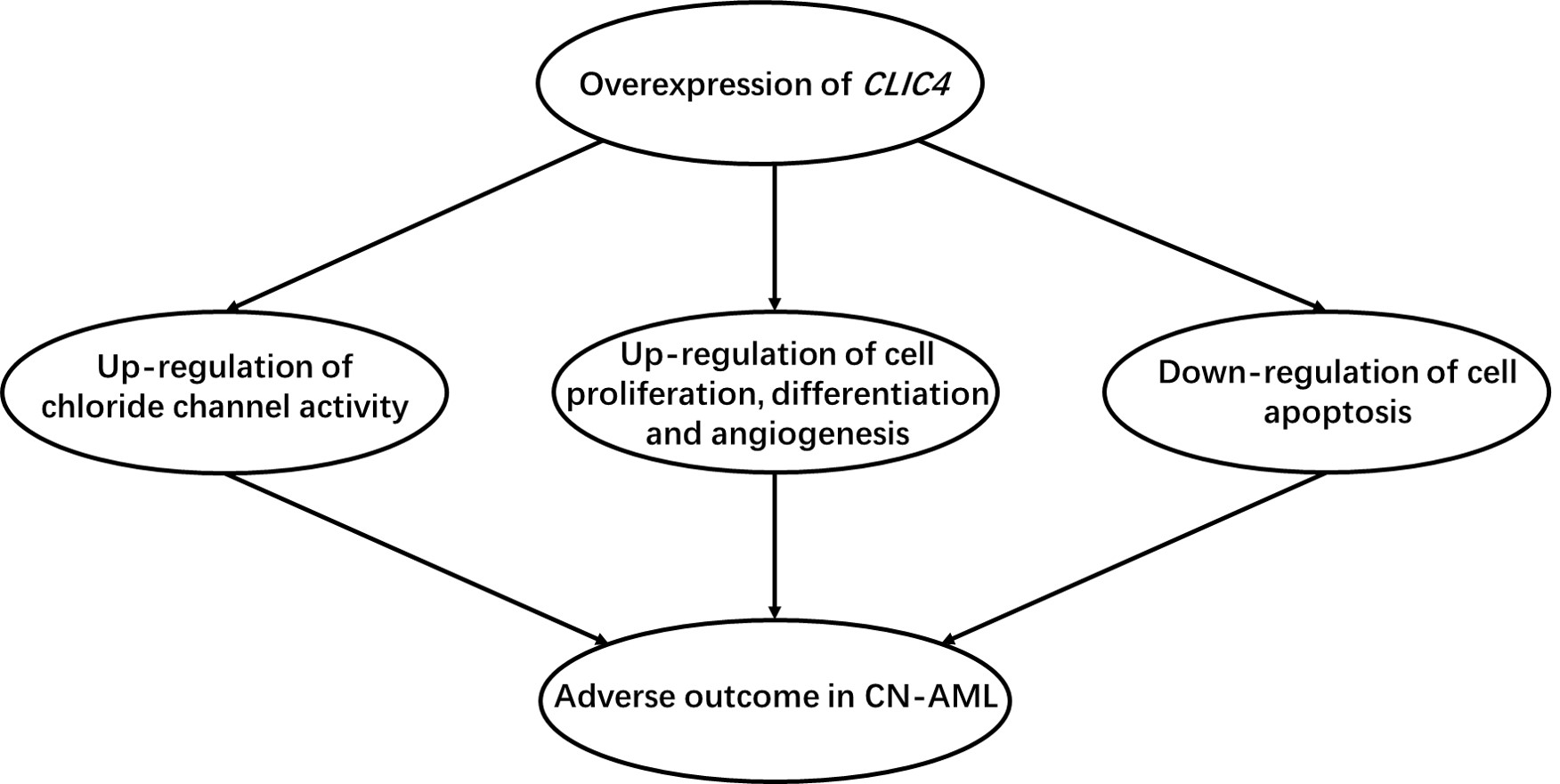

Supplement: FIGURE S3 — The hypothetical mechanism figure of CLIC4 in CN-AML. [file Image_3.JPEG]
