# Supplementary material for: Aberrant Chloride Intracellular Channel 4 Expression Is Associated With Adverse Outcome in Cytogenetically Normal Acute Myeloid Leukemia
Source: Front Oncol. 2020 Sep 9;10:1648. doi: 10.3389/fonc.2020.01648 (PMC7507859; doi:10.3389/fonc.2020.01648)
Supplement: Supplementary file 5 [file Data_Sheet_2.docx]

Table S1 105 genes significantly enriched in chronic lymphocytic leukemia (CLL)

| No. | Entrez Gene ID | Gene Symbol | Gene Name |
| --- | --- | --- | --- |
| 1 | 2072 | ERCC4 | ERCC excision repair 4, endonuclease catalytic subunit |
| 2 | 4121 | MAN1A1 | mannosidase alpha class 1A member 1 |
| 3 | 6198 | RPS6KB1 | ribosomal protein S6 kinase B1 |
| 4 | 53335 | BCL11A | BAF chromatin remodeling complex subunit BCL11A |
| 5 | 4189 | DNAJB9 | DnaJ heat shock protein family (Hsp40) member B9 |
| 6 | 51320 | MEX3C | mex-3 RNA binding family member C |
| 7 | 51360 | MBTPS2 | membrane bound transcription factor peptidase, site 2 |
| 8 | 57508 | INTS2 | integrator complex subunit 2 |
| 9 | 51439 | FAM8A1 | family with sequence similarity 8 member A1 |
| 10 | 57609 | DIP2B | disco interacting protein 2 homolog B |
| 11 | 55591 | VEZT | vezatin, adherens junctions transmembrane protein |
| 12 | 22841 | RAB11FIP2 | RAB11 family interacting protein 2 |
| 13 | 10560 | SLC19A2 | solute carrier family 19 member 2 |
| 14 | 55632 | G2E3 | G2/M-phase specific E3 ubiquitin protein ligase |
| 15 | 22887 | FOXJ3 | forkhead box J3 |
| 16 | 22890 | ZBTB1 | zinc finger and BTB domain containing 1 |
| 17 | 22894 | DIS3 | DIS3 homolog, exosome endoribonuclease and 3'-5' exoribonuclease |
| 18 | 51569 | UFM1 | ubiquitin fold modifier 1 |
| 19 | 57721 | METTL14 | methyltransferase like 14 |
| 20 | 22911 | WDR47 | WD repeat domain 47 |
| 21 | 10640 | EXOC5 | exocyst complex component 5 |
| 22 | 22929 | SEPHS1 | selenophosphate synthetase 1 |
| 23 | 246175 | CNOT6L | CCR4-NOT transcription complex subunit 6 like |
| 24 | 6558 | SLC12A2 | solute carrier family 12 member 2 |
| 25 | 6566 | SLC16A1 | solute carrier family 16 member 1 |
| 26 | 65977 | PLEKHA3 | pleckstrin homology domain containing A3 |
| 27 | 6596 | HLTF | helicase like transcription factor |
| 28 | 27086 | FOXP1 | forkhead box P1 |
| 29 | 147929 | ZNF565 | zinc finger protein 565 |
| 30 | 481 | ATP1B1 | ATPase Na+/K+ transporting subunit beta 1 |
| 31 | 23032 | USP33 | ubiquitin specific peptidase 33 |
| 32 | 2553 | GABPB1 | GA binding protein transcription factor subunit beta 1 |
| 33 | 23041 | MON2 | MON2 homolog, regulator of endosome-to-Golgi trafficking |
| 34 | 546 | ATRX | ATRX chromatin remodeler |
| 35 | 4664 | NAB1 | NGFI-A binding protein 1 |
| 36 | 8766 | RAB11A | RAB11A, member RAS oncogene family |
| 37 | 6747 | SSR3 | signal sequence receptor subunit 3 |
| 38 | 27248 | ERLEC1 | endoplasmic reticulum lectin 1 |
| 39 | 55930 | MYO5C | myosin VC |
| 40 | 6782 | HSPA13 | heat shock protein family A (Hsp70) member 13 |
| 41 | 6788 | STK3 | serine/threonine kinase 3 |
| 42 | 2744 | GLS | glutaminase |
| 43 | 8899 | PRPF4B | pre-mRNA processing factor 4B |
| 44 | 2776 | GNAQ | G protein subunit alpha q |
| 45 | 84725 | PLEKHA8 | pleckstrin homology domain containing A8 |
| 46 | 6938 | TCF12 | transcription factor 12 |
| 47 | 56180 | MOSPD1 | motile sperm domain containing 1 |
| 48 | 2957 | GTF2A1 | general transcription factor IIA subunit 1 |
| 49 | 7072 | TIA1 | TIA1 cytotoxic granule associated RNA binding protein |
| 50 | 5048 | PAFAH1B1 | platelet activating factor acetylhydrolase 1b regulatory subunit 1 |
| 51 | 23484 | LEPROTL1 | leptin receptor overlapping transcript like 1 |
| 52 | 7110 | TMF1 | TATA element modulatory factor 1 |
| 53 | 9175 | MAP3K13 | mitogen-activated protein kinase kinase kinase 13 |
| 54 | 152559 | PAQR3 | progestin and adipoQ receptor family member 3 |
| 55 | 283635 | FAM177A1 | family with sequence similarity 177 member A1 |
| 56 | 7163 | TPD52 | tumor protein D52 |
| 57 | 9255 | AIMP1 | aminoacyl tRNA synthetase complex interacting multifunctional protein 1 |
| 58 | 23592 | LEMD3 | LEM domain containing 3 |
| 59 | 1073 | CFL2 | cofilin 2 |
| 60 | 11320 | MGAT4A | alpha-1,3-mannosyl-glycoprotein 4-beta-N-acetylglucosaminyltransferase A |
| 61 | 60485 | SAV1 | salvador family WW domain containing protein 1 |
| 62 | 3189 | HNRNPH3 | heterogeneous nuclear ribonucleoprotein H3 |
| 63 | 79003 | MIS12 | MIS12 kinetochore complex component |
| 64 | 9397 | NMT2 | N-myristoyltransferase 2 |
| 65 | 54467 | ANKIB1 | ankyrin repeat and IBR domain containing 1 |
| 66 | 54468 | MIOS | meiosis regulator for oocyte development |
| 67 | 9534 | ZNF254 | zinc finger protein 254 |
| 68 | 56650 | CLDND1 | claudin domain containing 1 |
| 69 | 25932 | CLIC4 | chloride intracellular channel 4 |
| 70 | 64854 | USP46 | ubiquitin specific peptidase 46 |
| 71 | 7525 | YES1 | YES proto-oncogene 1, Src family tyrosine kinase |
| 72 | 5494 | PPM1A | protein phosphatase, Mg2+/Mn2+ dependent 1A |
| 73 | 54665 | RSBN1 | round spermatid basic protein 1 |
| 74 | 114088 | TRIM9 | tripartite motif containing 9 |
| 75 | 54704 | PDP1 | pyruvate dehyrogenase phosphatase catalytic subunit 1 |
| 76 | 5567 | PRKACB | protein kinase cAMP-activated catalytic subunit beta |
| 77 | 5584 | PRKCI | protein kinase C iota |
| 78 | 26091 | HERC4 | HECT and RLD domain containing E3 ubiquitin protein ligase 4 |
| 79 | 9743 | ARHGAP32 | Rho GTPase activating protein 32 |
| 80 | 253512 | SLC25A30 | solute carrier family 25 member 30 |
| 81 | 5716 | PSMD10 | proteasome 26S subunit, non-ATPase 10 |
| 82 | 54874 | FNBP1L | formin binding protein 1 like |
| 83 | 142940 | TRUB1 | TruB pseudouridine synthase family member 1 |
| 84 | 124540 | MSI2 | musashi RNA binding protein 2 |
| 85 | 81550 | TDRD3 | tudor domain containing 3 |
| 86 | 7844 | RNF103 | ring finger protein 103 |
| 87 | 9908 | G3BP2 | G3BP stress granule assembly factor 2 |
| 88 | 1739 | DLG1 | discs large MAGUK scaffold protein 1 |
| 89 | 286410 | ATP11C | ATPase phospholipid transporting 11C |
| 90 | 120526 | DNAJC24 | DnaJ heat shock protein family (Hsp40) member C24 |
| 91 | 83699 | SH3BGRL2 | SH3 domain binding glutamate rich protein like 2 |
| 92 | 5884 | RAD17 | RAD17 checkpoint clamp loader component |
| 93 | 79634 | SCRN3 | secernin 3 |
| 94 | 5911 | RAP2A | RAP2A, member of RAS oncogene family |
| 95 | 1859 | DYRK1A | dual specificity tyrosine phosphorylation regulated kinase 1A |
| 96 | 55110 | MAGOHB | mago homolog B, exon junction complex subunit |
| 97 | 55120 | FANCL | FA complementation group L |
| 98 | 8034 | SLC25A16 | solute carrier family 25 member 16 |
| 99 | 55156 | ARMC1 | armadillo repeat containing 1 |
| 100 | 8065 | CUL5 | cullin 5 |
| 101 | 221079 | ARL5B | ADP ribosylation factor like GTPase 5B |
| 102 | 3998 | LMAN1 | lectin, mannose binding 1 |
| 103 | 51141 | INSIG2 | insulin induced gene 2 |
| 104 | 135112 | NCOA7 | nuclear receptor coactivator 7 |
| 105 | 4094 | MAF | MAF bZIP transcription factor |

Table S2 153 genes significantly enriched in CD34+ cells isolated from bone marrow of patients with chronic myeloid leukemia (CML)

| No. | Entrez Gene ID | Gene Symbol | Gene Name |
| --- | --- | --- | --- |
| 1 | 10240 | MRPS31 | mitochondrial ribosomal protein S31 |
| 2 | 2060 | EPS15 | epidermal growth factor receptor pathway substrate 15 |
| 3 | 48 | ACO1 | aconitase 1 |
| 4 | 52 | ACP1 | acid phosphatase 1 |
| 5 | 10314 | LANCL1 | LanC like 1 |
| 6 | 2149 | F2R | coagulation factor II thrombin receptor |
| 7 | 2150 | F2RL1 | F2R like trypsin receptor 1 |
| 8 | 53371 | NUP54 | nucleoporin 54 |
| 9 | 128 | ADH5 | alcohol dehydrogenase 5 (class III), chi polypeptide |
| 10 | 6319 | SCD | stearoyl-CoA desaturase |
| 11 | 10427 | SEC24B | SEC24 homolog B, COPII coat complex component |
| 12 | 51439 | FAM8A1 | family with sequence similarity 8 member A1 |
| 13 | 51441 | YTHDF2 | YTH N6-methyladenosine RNA binding protein 2 |
| 14 | 8450 | CUL4B | cullin 4B |
| 15 | 4361 | MRE11 | MRE11 homolog, double strand break repair nuclease |
| 16 | 6426 | SRSF1 | serine and arginine rich splicing factor 1 |
| 17 | 10527 | IPO7 | importin 7 |
| 18 | 22872 | SEC31A | SEC31 homolog A, COPII coat complex component |
| 19 | 8545 | CGGBP1 | CGG triplet repeat binding protein 1 |
| 20 | 10605 | PAIP1 | poly(A) binding protein interacting protein 1 |
| 21 | 6526 | SLC5A3 | solute carrier family 5 member 3 |
| 22 | 22916 | NCBP2 | nuclear cap binding protein subunit 2 |
| 23 | 399 | RHOH | ras homolog family member H |
| 24 | 400 | ARL1 | ADP ribosylation factor like GTPase 1 |
| 25 | 10640 | EXOC5 | exocyst complex component 5 |
| 26 | 22929 | SEPHS1 | selenophosphate synthetase 1 |
| 27 | 29079 | MED4 | mediator complex subunit 4 |
| 28 | 6566 | SLC16A1 | solute carrier family 16 member 1 |
| 29 | 80321 | CEP70 | centrosomal protein 70 |
| 30 | 6596 | HLTF | helicase like transcription factor |
| 31 | 10728 | PTGES3 | prostaglandin E synthase 3 |
| 32 | 8723 | SNX4 | sorting nexin 4 |
| 33 | 10771 | ZMYND11 | zinc finger MYND-type containing 11 |
| 34 | 10776 | ARPP19 | cAMP regulated phosphoprotein 19 |
| 35 | 2589 | GALNT1 | polypeptide N-acetylgalactosaminyltransferase 1 |
| 36 | 8738 | CRADD | CASP2 and RIPK1 domain containing adaptor with death domain |
| 37 | 546 | ATRX | ATRX chromatin remodeler |
| 38 | 2620 | GAS2 | growth arrest specific 2 |
| 39 | 64062 | RBM26 | RNA binding motif protein 26 |
| 40 | 8766 | RAB11A | RAB11A, member RAS oncogene family |
| 41 | 6729 | SRP54 | signal recognition particle 54 |
| 42 | 586 | BCAT1 | branched chain amino acid transaminase 1 |
| 43 | 4690 | NCK1 | NCK adaptor protein 1 |
| 44 | 594 | BCKDHB | branched chain keto acid dehydrogenase E1 subunit beta |
| 45 | 64083 | GOLPH3 | golgi phosphoprotein 3 |
| 46 | 6745 | SSR1 | signal sequence receptor subunit 1 |
| 47 | 4698 | NDUFA5 | NADH:ubiquinone oxidoreductase subunit A5 |
| 48 | 55900 | ZNF302 | zinc finger protein 302 |
| 49 | 8803 | SUCLA2 | succinate-CoA ligase ADP-forming subunit beta |
| 50 | 6760 | SS18 | SS18 subunit of BAF chromatin remodeling complex |
| 51 | 10857 | PGRMC1 | progesterone receptor membrane component 1 |
| 52 | 8813 | DPM1 | dolichyl-phosphate mannosyltransferase subunit 1, catalytic |
| 53 | 6775 | STAT4 | signal transducer and activator of transcription 4 |
| 54 | 6782 | HSPA13 | heat shock protein family A (Hsp70) member 13 |
| 55 | 8835 | SOCS2 | suppressor of cytokine signaling 2 |
| 56 | 8848 | TSC22D1 | TSC22 domain family member 1 |
| 57 | 10920 | COPS8 | COP9 signalosome subunit 8 |
| 58 | 2739 | GLO1 | glyoxalase I |
| 59 | 10933 | MORF4L1 | mortality factor 4 like 1 |
| 60 | 10944 | C11orf58 | chromosome 11 open reading frame 58 |
| 61 | 4801 | NFYB | nuclear transcription factor Y subunit beta |
| 62 | 10959 | TMED2 | transmembrane p24 trafficking protein 2 |
| 63 | 2776 | GNAQ | G protein subunit alpha q |
| 64 | 23256 | SCFD1 | sec1 family domain containing 1 |
| 65 | 10982 | MAPRE2 | microtubule associated protein RP/EB family member 2 |
| 66 | 10983 | CCNI | cyclin I |
| 67 | 10987 | COPS5 | COP9 signalosome subunit 5 |
| 68 | 6938 | TCF12 | transcription factor 12 |
| 69 | 27430 | MAT2B | methionine adenosyltransferase 2B |
| 70 | 11051 | NUDT21 | nudix hydrolase 21 |
| 71 | 23369 | PUM2 | pumilio RNA binding family member 2 |
| 72 | 6996 | TDG | thymine DNA glycosylase |
| 73 | 9053 | MAP7 | microtubule associated protein 7 |
| 74 | 7013 | TERF1 | telomeric repeat binding factor 1 |
| 75 | 2926 | GRSF1 | G-rich RNA sequence binding factor 1 |
| 76 | 900 | CCNG1 | cyclin G1 |
| 77 | 5000 | ORC4 | origin recognition complex subunit 4 |
| 78 | 23435 | TARDBP | TAR DNA binding protein |
| 79 | 2956 | MSH6 | mutS homolog 6 |
| 80 | 7072 | TIA1 | TIA1 cytotoxic granule associated RNA binding protein |
| 81 | 11168 | PSIP1 | PC4 and SFRS1 interacting protein 1 |
| 82 | 11183 | MAP4K5 | mitogen-activated protein kinase kinase kinase kinase 5 |
| 83 | 961 | CD47 | CD47 molecule |
| 84 | 966 | CD59 | CD59 molecule (CD59 blood group) |
| 85 | 9166 | EBAG9 | estrogen receptor binding site associated antigen 9 |
| 86 | 988 | CDC5L | cell division cycle 5 like |
| 87 | 3075 | CFH | complement factor H |
| 88 | 23556 | PIGN | phosphatidylinositol glycan anchor biosynthesis class N |
| 89 | 9240 | PNMA1 | PNMA family member 1 |
| 90 | 23588 | KLHDC2 | kelch domain containing 2 |
| 91 | 9255 | AIMP1 | aminoacyl tRNA synthetase complex interacting multifunctional protein 1 |
| 92 | 1070 | CETN3 | centrin 3 |
| 93 | 5168 | ENPP2 | ectonucleotide pyrophosphatase/phosphodiesterase 2 |
| 94 | 60485 | SAV1 | salvador family WW domain containing protein 1 |
| 95 | 60496 | AASDHPPT | aminoadipate-semialdehyde dehydrogenase-phosphopantetheinyl transferase |
| 96 | 58478 | ENOPH1 | enolase-phosphatase 1 |
| 97 | 1154 | CISH | cytokine inducible SH2 containing protein |
| 98 | 23683 | PRKD3 | protein kinase D3 |
| 99 | 1161 | ERCC8 | ERCC excision repair 8, CSA ubiquitin ligase complex subunit |
| 100 | 5270 | SERPINE2 | serpin family E member 2 |
| 101 | 5292 | PIM1 | Pim-1 proto-oncogene, serine/threonine kinase |
| 102 | 7342 | UBP1 | upstream binding protein 1 |
| 103 | 9397 | NMT2 | N-myristoyltransferase 2 |
| 104 | 29883 | CNOT7 | CCR4-NOT transcription complex subunit 7 |
| 105 | 23741 | EID1 | EP300 interacting inhibitor of differentiation 1 |
| 106 | 7360 | UGP2 | UDP-glucose pyrophosphorylase 2 |
| 107 | 9440 | MED17 | mediator complex subunit 17 |
| 108 | 64746 | ACBD3 | acyl-CoA binding domain containing 3 |
| 109 | 5359 | PLSCR1 | phospholipid scramblase 1 |
| 110 | 7444 | VRK2 | VRK serine/threonine kinase 2 |
| 111 | 9520 | NPEPPS | aminopeptidase puromycin sensitive |
| 112 | 56650 | CLDND1 | claudin domain containing 1 |
| 113 | 25932 | CLIC4 | chloride intracellular channel 4 |
| 114 | 7514 | XPO1 | exportin 1 |
| 115 | 7525 | YES1 | YES proto-oncogene 1, Src family tyrosine kinase |
| 116 | 7528 | YY1 | YY1 transcription factor |
| 117 | 5494 | PPM1A | protein phosphatase, Mg2+/Mn2+ dependent 1A |
| 118 | 5516 | PPP2CB | protein phosphatase 2 catalytic subunit beta |
| 119 | 7570 | ZNF22 | zinc finger protein 22 |
| 120 | 7572 | ZNF24 | zinc finger protein 24 |
| 121 | 5529 | PPP2R5E | protein phosphatase 2 regulatory subunit B'epsilon |
| 122 | 5537 | PPP6C | protein phosphatase 6 catalytic subunit |
| 123 | 9637 | FEZ2 | fasciculation and elongation protein zeta 2 |
| 124 | 1457 | CSNK2A1 | casein kinase 2 alpha 1 |
| 125 | 9653 | HS2ST1 | heparan sulfate 2-O-sulfotransferase 1 |
| 126 | 5567 | PRKACB | protein kinase cAMP-activated catalytic subunit beta |
| 127 | 9698 | PUM1 | pumilio RNA binding family member 1 |
| 128 | 9782 | MATR3 | matrin 3 |
| 129 | 7756 | ZNF207 | zinc finger protein 207 |
| 130 | 5716 | PSMD10 | proteasome 26S subunit, non-ATPase 10 |
| 131 | 56938 | ARNTL2 | aryl hydrocarbon receptor nuclear translocator like 2 |
| 132 | 7812 | CSDE1 | cold shock domain containing E1 |
| 133 | 9874 | TLK1 | tousled like kinase 1 |
| 134 | 26275 | HIBCH | 3-hydroxyisobutyryl-CoA hydrolase |
| 135 | 81579 | PLA2G12A | phospholipase A2 group XIIA |
| 136 | 5810 | RAD1 | RAD1 checkpoint DNA exonuclease |
| 137 | 9908 | G3BP2 | G3BP stress granule assembly factor 2 |
| 138 | 5884 | RAD17 | RAD17 checkpoint clamp loader component |
| 139 | 57092 | PCNP | PEST proteolytic signal containing nuclear protein |
| 140 | 3845 | KRAS | KRAS proto-oncogene, GTPase |
| 141 | 5898 | RALA | RAS like proto-oncogene A |
| 142 | 10006 | ABI1 | abl interactor 1 |
| 143 | 81689 | ISCA1 | iron-sulfur cluster assembly 1 |
| 144 | 5928 | RBBP4 | RB binding protein 4, chromatin remodeling factor |
| 145 | 3895 | KTN1 | kinectin 1 |
| 146 | 1859 | DYRK1A | dual specificity tyrosine phosphorylation regulated kinase 1A |
| 147 | 8065 | CUL5 | cullin 5 |
| 148 | 3998 | LMAN1 | lectin, mannose binding 1 |
| 149 | 1964 | EIF1AX | eukaryotic translation initiation factor 1A X-linked |
| 150 | 10179 | RBM7 | RNA binding motif protein 7 |
| 151 | 51185 | CRBN | cereblon |
| 152 | 4089 | SMAD4 | SMAD family member 4 |
| 153 | 10236 | HNRNPR | heterogeneous nuclear ribonucleoprotein R |

Table S3 53 genes significantly up-regulated in cancer stem cells isolated from mammary tumors

| No. | Entrez Gene ID | Gene Symbol | Gene Name |
| --- | --- | --- | --- |
| 1 | 10240 | MRPS31 | mitochondrial ribosomal protein S31 |
| 2 | 10282 | BET1 | Bet1 golgi vesicular membrane trafficking protein |
| 3 | 51247 | PAIP2 | poly(A) binding protein interacting protein 2 |
| 4 | 53335 | BCL11A | BAF chromatin remodeling complex subunit BCL11A |
| 5 | 57472 | CNOT6 | CCR4-NOT transcription complex subunit 6 |
| 6 | 128 | ADH5 | alcohol dehydrogenase 5 (class III), chi polypeptide |
| 7 | 57508 | INTS2 | integrator complex subunit 2 |
| 8 | 84135 | UTP15 | UTP15 small subunit processome component |
| 9 | 57531 | HACE1 | HECT domain and ankyrin repeat containing E3 ubiquitin protein ligase 1 |
| 10 | 10513 | APPBP2 | amyloid beta precursor protein binding protein 2 |
| 11 | 51496 | CTDSPL2 | CTD small phosphatase like 2 |
| 12 | 28970 | C11orf54 | chromosome 11 open reading frame 54 |
| 13 | 28981 | IFT81 | intraflagellar transport 81 |
| 14 | 51542 | VPS54 | VPS54 subunit of GARP complex |
| 15 | 55728 | N4BP2 | NEDD4 binding protein 2 |
| 16 | 84455 | EFCAB7 | EF-hand calcium binding domain 7 |
| 17 | 4591 | TRIM37 | tripartite motif containing 37 |
| 18 | 23047 | PDS5B | PDS5 cohesin associated factor B |
| 19 | 10776 | ARPP19 | cAMP regulated phosphoprotein 19 |
| 20 | 8731 | RNMT | RNA guanine-7 methyltransferase |
| 21 | 596 | BCL2 | BCL2 apoptosis regulator |
| 22 | 8803 | SUCLA2 | succinate-CoA ligase ADP-forming subunit beta |
| 23 | 8883 | NAE1 | NEDD8 activating enzyme E1 subunit 1 |
| 24 | 4801 | NFYB | nuclear transcription factor Y subunit beta |
| 25 | 10971 | YWHAQ | tyrosine 3-monooxygenase/tryptophan 5-monooxygenase activation protein theta |
| 26 | 2824 | GPM6B | glycoprotein M6B |
| 27 | 780 | DDR1 | discoidin domain receptor tyrosine kinase 1 |
| 28 | 129831 | RBM45 | RNA binding motif protein 45 |
| 29 | 64431 | ACTR6 | actin related protein 6 |
| 30 | 1070 | CETN3 | centrin 3 |
| 31 | 128178 | EDARADD | EDAR associated death domain |
| 32 | 23741 | EID1 | EP300 interacting inhibitor of differentiation 1 |
| 33 | 9406 | ZRANB2 | zinc finger RANBP2-type containing 2 |
| 34 | 54556 | ING3 | inhibitor of growth family member 3 |
| 35 | 728340 | GTF2H2C | GTF2H2 family member C |
| 36 | 25913 | POT1 | protection of telomeres 1 |
| 37 | 132430 | PABPC4L | poly(A) binding protein cytoplasmic 4 like |
| 38 | 7514 | XPO1 | exportin 1 |
| 39 | 7570 | ZNF22 | zinc finger protein 22 |
| 40 | 7572 | ZNF24 | zinc finger protein 24 |
| 41 | 26053 | AUTS2 | activator of transcription and developmental regulator AUTS2 |
| 42 | 9678 | PHF14 | PHD finger protein 14 |
| 43 | 7678 | ZNF124 | zinc finger protein 124 |
| 44 | 26127 | FGFR1OP2 | FGFR1 oncogene partner 2 |
| 45 | 7707 | ZNF148 | zinc finger protein 148 |
| 46 | 9782 | MATR3 | matrin 3 |
| 47 | 3673 | ITGA2 | integrin subunit alpha 2 |
| 48 | 9868 | TOMM70 | translocase of outer mitochondrial membrane 70 |
| 49 | 284307 | ZIK1 | zinc finger protein interacting with K protein 1 |
| 50 | 9908 | G3BP2 | G3BP stress granule assembly factor 2 |
| 51 | 1787 | TRDMT1 | tRNA aspartic acid methyltransferase 1 |
| 52 | 57181 | SLC39A10 | solute carrier family 39 member 10 |
| 53 | 55251 | PCMTD2 | protein-L-isoaspartate (D-aspartate) O-methyltransferase domain containing 2 |

Table S4 19 genes significantly enriched in a probe-set gene-expression signature that predicted survival in CN-AML

| No. | Entrez Gene ID | Gene Symbol | Gene Name |
| --- | --- | --- | --- |
| 1 | 4130 | MAP1A | microtubule associated protein 1A |
| 2 | 135228 | CD109 | CD109 molecule |
| 3 | 53335 | BCL11A | BAF chromatin remodeling complex subunit BCL11A |
| 4 | 51309 | ARMCX1 | armadillo repeat containing X-linked 1 |
| 5 | 6319 | SCD | stearoyl-CoA desaturase |
| 6 | 2273 | FHL1 | four and a half LIM domains 1 |
| 7 | 6678 | SPARC | secreted protein acidic and cysteine rich |
| 8 | 586 | BCAT1 | branched chain amino acid transaminase 1 |
| 9 | 55930 | MYO5C | myosin VC |
| 10 | 8835 | SOCS2 | suppressor of cytokine signaling 2 |
| 11 | 8848 | TSC22D1 | TSC22 domain family member 1 |
| 12 | 375449 | MAST4 | microtubule associated serine/threonine kinase family member 4 |
| 13 | 861 | RUNX1 | RUNX family transcription factor 1 |
| 14 | 83543 | AIF1L | allograft inflammatory factor 1 like |
| 15 | 124540 | MSI2 | musashi RNA binding protein 2 |
| 16 | 255631 | COL24A1 | collagen type XXIV alpha 1 chain |
| 17 | 653121 | ZBTB8A | zinc finger and BTB domain containing 8A |
| 18 | 10152 | ABI2 | abl interactor 2 |
| 19 | 4071 | TM4SF1 | transmembrane 4 L six family member 1 |

Table S5 23 genes among top 100 probe sets for pediatric AML subtypes with chimeric MLL fusions

|  | Entrez Gene ID | Gene Symbol | Gene Name |
| --- | --- | --- | --- |
| 1 | 3101 | HK3 | hexokinase 3 |
| 2 | 51324 | SPG21 | SPG21 abhydrolase domain containing, maspardin |
| 3 | 1200 | TPP1 | tripeptidyl peptidase 1 |
| 4 | 2242 | FES | FES proto-oncogene, tyrosine kinase |
| 5 | 221 | ALDH3B1 | aldehyde dehydrogenase 3 family member B1 |
| 6 | 6368 | CCL23 | C-C motif chemokine ligand 23 |
| 7 | 9450 | LY86 | lymphocyte antigen 86 |
| 8 | 241 | ALOX5AP | arachidonate 5-lipoxygenase activating protein |
| 9 | 64780 | MICAL1 | microtubule associated monooxygenase, calponin and LIM domain containing 1 |
| 10 | 1298 | COL9A2 | collagen type IX alpha 2 chain |
| 11 | 344 | APOC2 | apolipoprotein C2 |
| 12 | 29108 | PYCARD | PYD and CARD domain containing |
| 13 | 2629 | GBA | glucosylceramidase beta |
| 14 | 2647 | BLOC1S1 | biogenesis of lysosomal organelles complex 1 subunit 1 |
| 15 | 3687 | ITGAX | integrin subunit alpha X |
| 16 | 6813 | STXBP2 | syntaxin binding protein 2 |
| 17 | 9961 | MVP | major vault protein |
| 18 | 822 | CAPG | capping actin protein, gelsolin like |
| 19 | 3956 | LGALS1 | galectin 1 |
| 20 | 6037 | RNASE3 | ribonuclease A family member 3 |
| 21 | 6050 | RNH1 | ribonuclease/angiogenin inhibitor 1 |
| 22 | 2990 | GUSB | glucuronidase beta |
| 23 | 59342 | SCPEP1 | serine carboxypeptidase 1 |
